# *FCGR2A*-HH Gene Variants Encoding the Fc Gamma Receptor for the C-Reactive Protein Are Associated with Enhanced Monocyte CD32 Expression and Cardiovascular Events’ Recurrence after Primary Acute Coronary Syndrome

**DOI:** 10.3390/biomedicines10020495

**Published:** 2022-02-19

**Authors:** Pascale Paul, Christophe Picard, Luc Lyonnet, Noémie Resseguier, Lucas Hubert, Laurent Arnaud, Julie Di Cristofaro, Marc Laine, Franck Paganelli, Françoise Dignat-George, Corinne Frère, Florence Sabatier, Regis Guieu, Laurent Bonello

**Affiliations:** 1 INSERM 1263, Aix Marseille Université, INRAE, 13005 Marseille, France; francoise.dignat.george@univ-amu.fr (F.D.-G.); florence.sabatier@ap-hm.fr (F.S.); regis.guieu@ap-hm.fr (R.G.); laurent.bonello@ap-hm.fr (L.B.); 2 Department of Hematology, Hopital de la Conception, Assistance Publique-Hôpitaux Marseille, 13005 Marseille, France; luc.lyonnet@ap-hm.fr (L.L.); laurent.arnaud@ap-hm.fr (L.A.); 3 INSERM UMR_1090, Aix Marseille Université, TAGC Theories and Approaches of Genomic Complexity, Institut MarMaRa, Parc Scientifique de Luminy Case 928, 163 Avenue de Luminy, CEDEX 09, 13288 Marseille, France; 4 Biologie des Groupes Sanguins, Établissement Français du Sang, UMR 7268 ADÉS EFS/CNRS, Aix-Marseille Université, 13005 Marseille, France; christophe.picard@efs.sante.fr (C.P.); lucas.hubert@efs.sante.fr (L.H.); julie.dicristofaro@efs.sante.fr (J.D.C.); 5 Support Unit for Clinical Research and Economic Evaluation, EA3279, CEReSS-Health Service Research and Quality of Life Center, Assistance Publique Hôpitaux de Marseille, 13005 Marseille, France; noemie.resseguier@univ-amu.fr; 6 Mediterranean Association for Research and Studies in Cardiology (MARS Cardio), 13015 Marseille, France; marc.laine@ap-hm.fr (M.L.); franck.paganelli@ap-hm.fr (F.P.); 7 Department of Cardiology, Assistance Publique-Hôpitaux de Marseille, Hôpital Nord, Aix-Marseille University, 13015 Marseille, France; 8 Institute of Cardiometabolism and Nutrition, GRC 27 GRECO, Sorbonne University, INSERM UMRS_1166, 75013 Paris, France; corinne.frere@aphp.fr; 9 Department of Biochemistry, Assistance Publique-Hôpitaux, 13005 Marseille, France

**Keywords:** Fc-gamma receptor polymorphism, cardiovascular inflammation, CD32, circulating monocytes, acute heart failure, MACE, C-reactive protein

## Abstract

Fcγ receptors (FcγRs) interact with the C-reactive protein (CRP) and mediate activation of inflammation-related pathogenic mechanisms affecting cardiovascular health. Our study evaluated whether FcγRIIA and FcγRIIIA profiles are associated with the recurrence of adverse cardiovascular events during the first year after a primary acute coronary syndrome (ACS). The primary endpoint was the recurrence of cardiovascular events (RCE), identified as a composite outcome comprising acute heart failure (AHF) and major adverse cardiovascular events (MACE). We obtained blood samples of 145 ACS patients to measure hsCRP circulating levels, to identify FcγRIIA-131RH rs1801274 and FcγRIIIA-158FV rs396991 polymorphisms, to analyze circulating monocytes and NK cell subsets expressing CD16 and CD32, and to detect serum-mediated FCGR2A-HH activation by luciferase reporter assays. The hsCRP, CD32-expression, and Fc-R mediated activation levels were similar in all patients regardless of their MACE risk. In contrast, the hsCRP levels and the proportion of CD14+ circulating monocytes expressing the CD32 receptor for CRP were significantly higher in the patients who developed AHF. The FCGR2A rs1801274 HH genotype was significantly more common in patients who developed RCE and MACE than in RCE-free patients and associated with an enhanced percentage of circulating CD32+CD14+ monocytes. The FCGR2A-HH genotype was identified as an independent predictor of subsequent RCE (OR, 2.7; *p* = 0.048; CI, 1.01–7.44) by multivariate analysis. These findings bring preliminary evidence that host FCGR2A genetic variants can influence monocyte CD32 receptor expression and may contribute to the fine-tuning of CD32-driven chronic activating signals that affect the risk of developing RCEs following primary ACS events.

## 1. Introduction

Patients undergoing percutaneous intervention (PCI) for acute coronary syndrome (ACS) are at high risk for recurrent adverse cardiovascular events. Various biomarkers that reflect physio-pathological inflammatory mechanisms underlying cardiac injury have been previously associated with the occurrence of ACS or the subsequent risk of major adverse cardiovascular events (MACE) [1,2,3]. Dysregulation of the inflammatory response is a major contributor to atherosclerosis progression and a target in the treatment of cardiovascular diseases (CVDs) [4,5]. Altered levels of circulating vascular dysfunction and inflammation factors, such as C-reactive protein (CRP), cytokines, and chemokines, have been reported in patients with ACS [6]. Elevated highly sensitive (hs) CRP has been suggested as a relevant biomarker to classify the inflammatory risk of patients [7], but mechanisms by which CRP expression is induced and activates signaling pathways that sustain CRP-driven inflammation and contribute to cardiovascular outcomes after PCI remain poorly understood. CRP has been shown to promote inflammatory signals resulting from its binding to the FcγRIIA/CD32 low-affinity receptor for IgG expressed on monocytes, neutrophils, and platelets. Various studies have characterized alteration of the FcγRIIA/CD32 and FcγRIIIA/CD16 activating FcγRs profiles in patients with cardiovascular diseases (reviewed in [8]). A s1801274 polymorphism in the corresponding *FCG2RA* gene (encoding the CD32 FcγRIIA low-affinity receptor for IgG and CRP) that results in an amino acid change at position 131 (histidine or arginine H131R) results in altered CRP- and IgG2-mediated signaling in monocytes and platelets [9,10]. In addition to platelets, neutrophils, and monocytes/macrophages, the FcγRIIA/CD32 receptor can be expressed by other cells that contribute to cardiovascular homeostasis, ischemic injury, and vascular dysfunction (such as endothelial cells [11,12] and cardiomyocytes [13]). Therefore, the CRP-CD32a signaling pathway might participate in inflammation-related pathogenic mechanisms associated with progression of cardiovascular and auto- and allo-immune disorders [14,15,16].The *FcγRIIIA-158 VV* genotype encoding the activating CD16 receptor, with a high affinity for the Fc fragment of IgG1 and IgG3, was also associated with a decreased risk of coronary artery disease and immune thrombocytopenia [17,18]. We aim to evaluate whether the presence of high hsCRP circulating levels, *FCGR2A*/*FCGR3A* polymorphisms, and their expression profiles could be associated with the occurrence of adverse cardiovascular events during the first year after PCI for ACS.

## 2. Material and Methods

### 2.1. Patients

We conducted a multicenter prospective observational pilot study of patients undergoing PCI for ACS (NCT02117037). All consecutive patients admitted to participating centers from September 2015 to September 2017 for intermediate, high-risk, or very high-risk ACS according to ESC guidelines were eligible to participate. Patients were included after coronary angiography if PCI was indicated. Exclusion criteria were as follows: age below 18 or over 80 years; pregnant or lactating women; concomitant diseases such as neoplasia, infection, or inflammatory diseases; antiplatelet therapy contraindications; cardiogenic shock; sustained ventricular arrhythmias; life expectancy lower than a year; and bleeding diathesis. Informed consents were obtained before inclusion. The study protocol conformed to the ethical guidelines of the 1975 Declaration of Helsinki and was approved by our institution’s human research committee in 2015. To compare frequencies of *FCGR2A* and *FCGR3A* genes between our patients and healthy individuals, who were representative of the southeastern France population in Marseille, we used blood samples from a previously described EFS blood bank control cohort of 208 healthy donors to compare frequencies of *FCGR2A* and *FCGR3A* genes between our patients and the healthy individuals that are representative of the southeastern France population in Marseille [15].

### 2.2. Follow-Up and Clinical Endpoint

All patients were followed up for 12 months after discharge. The primary endpoint was recurrence of cardiovascular events (RCE), defined as a composite endpoint of acute heart failure (AHF) and major adverse cardiovascular events (MACE) occurring during a 1-year post-PCI follow-up. Blood samples were obtained from 145 ACS patients. The secondary endpoint was AHF, defined as the acute presentation of clinical heart failure symptoms with elevated BNP levels or compatible echocardiographic findings with the need for diuretic treatment. A blinded committee of physicians adjudicated all events unaware of the study’s aim or biological results diagnosed.

### 2.3. Percutaneous Coronary Intervention

Procedural medications and coronary revascularizations were performed according to the ESC guidelines [19], at the discretion of each interventional cardiologist, and the approaches included staged procedures in some cases (Table 1). Whenever a PCI was considered appropriate, the medical researchers treated the culprit vessel in a single session. The same clinicians decided whether to perform complete revascularization in a single session or to treat patients with multivessel disease in a staged fashion.

### 2.4. Medication

All patients received an IV dose of 250 mg of aspirin upon admission for ACS. Patients with PCI indications received an oral loading dose of the P2Y12-ADP receptor antagonist. Either intravenous unfractionated heparin (UFH) or low molecular weight heparin (LMWH) was used during the PCI procedures. Any addition of glycoprotein IIB/IIIA inhibitors was at the discretion of the operator. Cangrelor was not available. All medications were used and dosed according to guidelines as soon as the ACS diagnosis was confirmed (Table 2).

### 2.5. Data Collection

Medical, biological, and angiographic data were retrieved from the electronic patient files. Hypertension was defined as a blood pressure of above 140/90 mmHg and/or being on antihypertensive drugs. Diabetes mellitus was defined according to the World Health Organization (WHO) criteria (fasting plasma glucose ≥ 7.0 mmol/L (126 mg/dL) or 2-h plasma glucose ≥ 11.1 mmol/L (200 mg/dL)), and/or being on anti-diabetic drugs. Anemia was defined at baseline as a serum hemoglobin < 12 g/dL in women (hematocrit < 36%) or <13 g/dL in men (hematocrit < 39%) according to the WHO criteria.

### 2.6. Blood Samples

All blood samples were collected at the time of PCIs for ACS. Peripheral blood mononuclear cells (PBMC) used for flow cytometry analysis of CD14 and lymphocyte cell subsets were isolated after density gradient centrifugation using lymphocyte separation media (Eurobio, Les Ulis, France) as previously described [20]. Serum samples were collected at the same time points during enrolment and 24 h after the PCI, and they were cryopreserved at −80 °C until later use to assess circulating levels of soluble CRP inflammatory marker.

### 2.7. Analysis of Soluble hsCRP Circulating Levels

All samples were analyzed in the central laboratory facilities of Timone University Hospital, Marseille, France. Hs C-reactive plasmatic protein concentrations were measured by turbidimetry using Cobas 8000 instrument (Roche Diagnosis, Meylan, France). The detection threshold was 0.3 mg/L, and the inter-assay coefficient of variation was <10%. We expressed results as milligrams per liter, and we set the detection limit and a 3 mg/L cut-off to classify patients as presenting either “low-grade peripheral inflammation” (≥3 mg/L) or “no peripheral inflammation” (<3 mg/L).

### 2.8. Flow Cytometry

We performed a phenotypic comparative analysis of CD16 and CD32 expressions in peripheral blood monocytes and NK cells in a subgroup of 24 RCE+ and RCE-free (control) patients, that were matched for age (±3 years), gender, and CKD status. A flow cytometry analysis of leucocyte cell surface receptor expressions was performed on cryopreserved PBMCs, after washing them in PBS (Gibco, Thermo Fisher Scientific, Waltham, MA, USA). A trypan blue staining was performed at the time of thawing to ensure the good viability of the cells. Cells were then incubated at 4 °C for 20 min with specified monoclonal antibodies. Following incubation and washing, we analyzed the samples in a Navios-3 lasers instrument (Beckman Coulter) using the Kaluza software. CD3+ lymphocytes and CD3-CD14+ monocyte and CD3-CD56+ NK cell subsets were gated among human PBMCs by flow cytometry using CD45-KromeOrange (Clone J33, Beckman Coulter, Brea, CA, USA), CD3-ECD (clone UCHT1, Beckman Coulter, Brea, CA, USA), CD56-PC7 (clone NKH-1, Beckman Coulter, Brea, CA, USA), and CD14-FITC (clone RMO52, Beckman Coulter, Brea, CA, USA). Briefly, lymphocyte and monocyte populations were recovered on a biexpo (dotplot) structure (SS)/CD45. Within the lymphocyte gate, CD3/CD56 staining allowed for further discrimination of the NK (CD3-CD56+) and T (CD3+) lymphocyte cell subsets. The CD14+ monocyte population was gated using Biexpo dotplot CD45/CD14. We detected the percentages of positive cells and their expression levels for Fc-gamma receptor markers using the following labeled antibodies: CD16-APC-AlexaFluor750 (clone 3G8, Beckman Coulter, Brea, CA, USA), CD32-APC (clone FLI8.26, BD Pharmingen, San Jose, CA, USA), and matched control isotypes.

### 2.9. Genotyping

We detected *FcγRIIA-*131RH rs1801274 and *FcγRIIIA-*158FV rs396991 genotypes by direct sequencing after PCR amplification as described [13,14]. Sequences were analyzed using the Codon Code Aligner program (Codon Code Corporation, Barnstable, MA, USA). Briefly, a multiplex PCR using primers flanking the target SNPs was performed on 100 ng of genomic DNA in a final volume of 25 μL containing PCR Qiagen Master Mix (Qiagen, Hilden, Germany). The second step consisted of a multiplex extension reaction performed using the SnapShot kit (Invitrogen, Thermo Fisher Scientific, Waltham, MA, USA) according to the manufacturer’s protocol. SnapShot extension primer data were analyzed using GeneMapper v4.0 with specific detection parameters.

### 2.10. Reporter Analysis of Serum-Mediated Activation of CD32-HH

The human *FcγRIIa* (H131 variant)-specific reporter bioassay complete kit (Promega; G9901) was used to quantify rh CRP/serum-dependent ADCP bioactivity [21]. The assay uses dual-engineered Jurkat effector cells that express the human *FcγRIIa* (H131 variant) receptor and mediate CD32-dependent NFAT activation of reporter luciferase expression upon binding of the receptors to their antibody ligand. We used rituximab, a chimeric IgG1 antibody drug with known ADCP activity through FcγRIIa, and Raji target cells as a positive control of the assay. Human C-reactive recombinant protein (rhCRP, ThermoFisher Scientific, RP-75528) produced in an *E. coli* expression system was supplied in solution at 1 mg/mL in TBS (pH 7.5 with 2 mM CaCl_2_). We used dilutions of rhCRP (400 mg/L, 100 mg/L, 25 mg/L, 6.25 mg/L, and 0 rhCRP) in the human AB serum of a non-immunized healthy male patient as a standard for the ADCP assay. Briefly, CRP or patients’ sera were incubated with Jurkat effector cells at 37 °C in a 5% CO_2_ environment for 6 h. Next, we added Bio-Glo™ Reagent and measured a bioluminescent signal using a Promega^®^ GloMax Explorer reader. We calculated values as means of two replicate relative light units (RLUs) that allowed for measurements of the fold induction in response to patient’s sera, when analyzed in reference to values measured in FCGR2A-HH-transfected Jurkat effector cells incubated in the presence of control human AB serum in the absence of added hsCRP. Fold induction was evaluated using the formula: RLU (serum induced − background)/RLU (no antibody control − background).

### 2.11. Statistical Analysis

Quantitative variables are described as means ± standard deviations or medians and quartiles (first quartile–third quartile) according to their distribution. Qualitative variables are described as numbers and percentages. We performed a bivariate analysis between occurrence of MACE/AHF/RCE and clinico-biological characteristics using a Student’s *t*-test for quantitative characteristics if appropriate (otherwise, we used a Mann–Whitney test), and a Chi-squared test for qualitative characteristics if appropriate (otherwise, we used a Fisher test). A *p* value < 0.05 was considered statistically significant for all tests. Correlations were assessed using the nonparametric Spearman’s rank correlation test. Receiver operating characteristic (ROC) curves were used to define the best threshold value for quantitative biomarkers and to discriminate patients with and without MACE, AHF, and RCE, according to the Youden’s method (which allows for the maximization of both sensitivity and specificity). A bivariate logistic regression allowed us to estimate crude odds ratios with their 95% confidence intervals (95% CI). In addition, we performed a multivariate logistic regression to assess the independent associations of clinico-biological characteristics with RCE. We selected relevant variables from the bivariate analysis, based on a threshold *p* value ≤ 0.1. Firth’s correction was applied by performing Firth’s penalized-likelihood logistic regression to consider small numbers. Adjusted odds ratios and their 95% confidence intervals were estimated. All tests were two sided and *p* < 0.05 was used to define statistical significance. All statistical analyses were performed using R software or GraphPad Prism 9.3 software (GraphPad Software Inc., San Diego, CA, USA) for the graphical representation of the figures.

## 3. RESULTS

### 3.1. Patients’ Characteristics

From September 2015 to September 2017, a total of 145 ACS patients undergoing PCI with a concomitant evaluation of baseline hsCRP circulating levels and analysis of *FcγRIIA-*131RH rs1801274 and *FcγRIIIA-*FV158 rs396991 polymorphic variants were enrolled in the study. The baseline characteristics of patients are shown in Table 2. At 1 year of clinical follow-up, 19% of patients experienced a major recurrent cardiovascular event (RCE, or a composite of MACE and/or AHF). From the 27 patients with at least one major recurrent cardiovascular event occurring during the first-year post enrolment, 18 patients experienced a MACE episode, also associated with AHF in 4 patients (AHF+MACE+), while 9 additional patients experienced an episode of AHF without MACE (13 patients, 9 AHF+MACE+ and 4 AHF+MACE− patients were thus reported in the AHF group, Table 2). We found that baseline anemia (OR, 2.7; *p* = 0.025; 95% CI, 1.13–6.25), chronic kidney disease (OR, 4.9; *p* = 0.029; 95% CI, 1.18–20.20), hypercholesterolemia (OR, 3.0; *p* = 0.010, CI: 1.3–7.14), and diabetes (OR, 2.6; *p* = 0.025; 95%CI: 1.13–6.25) at time of enrolment were significantly associated with RCE using univariate analysis. Gender or other classical cardiovascular risk factors at baseline (such as age, smoking, hypertension, and CVD history) did not significantly impact the risk of RCE recurrence. As we expected, LVEF values < 40 were identified as a risk factor for RCE (OR, 4.5; 95% CI, 1.56–12.69; *p* = 0.006). We also found higher baseline BNP (190 vs. 50.5; *p* = 0.0001), LDH levels (255 vs. 203; *p* = 0.044), and platelet counts (270 vs. 226.5; *p* = 0.0263), and lower creatinine clearance values (80 vs. 95; *p* = 0.006) and hemoglobin levels (124 vs. 135; *p* = 0.028) in patients who developed an RCE than in those who did not.

### 3.2. Baseline hsCRP Circulating Levels Are Associated with Subsequent AHF after PCI

**Figure 1:**
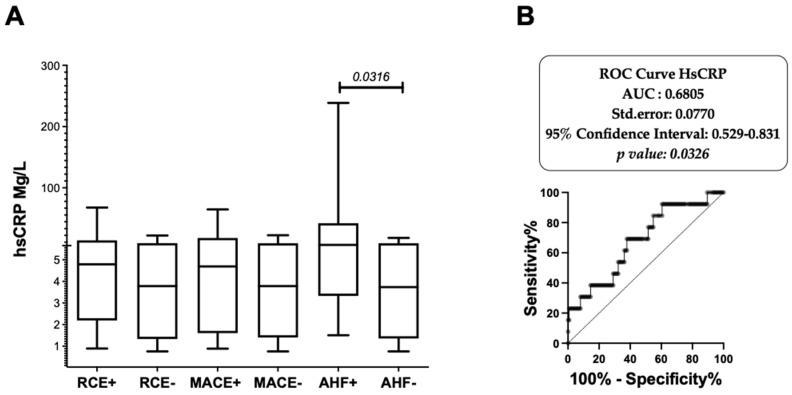
High circulating CRP levels are associated with AHF recurrence. (**A**) Comparative analysis of circulating hsCRP levels was analyzed in RCE (RCE+, *n* = 27) and non-RCE patients (RCE−, *n* = 118). HsCRP levels were then analyzed in the subgroups of patients experiencing MACE (MACE+ group, *n* = 18, among which 14 patients were MACE+AHF− and 4 patients experienced both MACE and AHF episodes during the first year post PCI) or AHF (AHF+ group, *n* = 13, 9 AHF+MACE− and 4 AHF+MACE+ patients) analyzed in reference to patients without MACE (MACE−, *n* = 127) or free of AHF (AHF−, *n* = 132) at one year post ACS. hsCRP variables are described in box and whiskers plots as medians and (10–90 percentile) using GraphPad Prism. Comparison between groups were performed using Mann–Whitney non-parametric unpaired *t* tests. *p* values < 0.05 were reported as significant. (**B**) ROC curve analysis of CRP levels associated with recurrent AHF episodes during the first year post initial ACS. Receiver operating characteristic (ROC) curves were used to define the best threshold value of hsCRP that allows us to discriminate patients with and without AHF, according to the Youden’s method (which allows for the maximization of both sensitivity and specificity).

The median baseline hsCRP level in the patient’s cohort was 3.97 mg/L (Figure 1). Baseline hsCRP levels were significantly correlated with the baseline levels of hemoglobin (*r* = 0.2366; *p* = 0.0054), fibrinogen (*r* = 0.655, *p* < 0.0001), LDH (*r* = 0.378, *p* < 0.0001), and BNP (*r* = 0.375, *p* < 0.0001), with platelet counts (*r* = 0.247; *p* = 0.005) and the percentage of CD16+ circulating monocytes (*r* = 0.349, *p* = 0.0343). In addition, the hsCRP levels were inversely correlated with the baseline LVEF (*r* = −0.2322; *p* = 0.0097) and CKD-EPI creatinine clearance (*r* = −0.2081; *p* = 0.0147) values. Circulating baseline hsCRP values did not differ between patients who further developed RCE and those who remained RCE free (Table 2), or between patients who further developed MACE (4.69 mg/L vs. 3.78 mg/L). In contrast, baseline hsCRP levels were significantly higher in patients who developed AHF than in the AHF-free control group (6.87 mg/L vs. 3.74 mg/L; *p* = 0.0329; see Figure 1A). ROC curve analysis (Figure 1B) allowed us to set a threshold for the hsCRP level at ≥2.6 mg/L (AUC, 0.6805; 95% IC, 0.5295–0.8315) to identify patients at high risk of AHR (OR, 5.46; 95% CI, 1.26–51.27; *p* = 0.0207, sensitivity 92.3%, specificity 39.52%, negative predictive value 98%, positive predictive value 14%).

**Figure 2:**
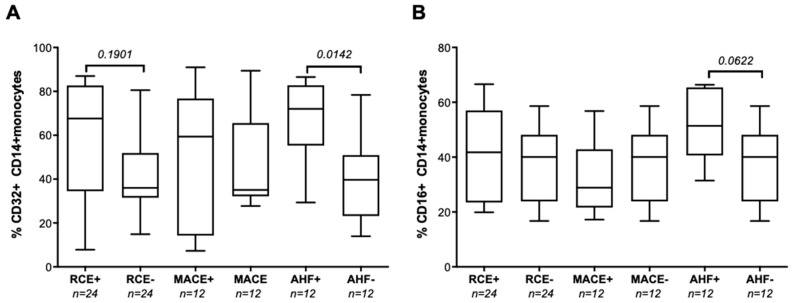
Flow cytometry analysis of circulating CD32+ and CD16+ monocyte. (**A**) Comparative analysis of circulating hsCRP levels was analyzed in RCE (RCE+, *n* = 27) and non-RCE patients (RCE−, *n* = 118). HsCRP levels were then analyzed in the subgroups of patients experiencing MACE (MACE+ group, *n* = 18, among which 14 patients were MACE+AHF− and 4 patients experienced both MACE and AHF episodes during the first year post PCI) or AHF (AHF+ group, *n* = 13, 9 AHF+MACE− and 4 AHF+MACE+ patients) analyzed in reference to patients without MACE (MACE−, *n* = 127) or free of AHF (AHF−, *n* = 132) at one year post ACS. hsCRP variables are described in box and whiskers plots as medians and (10–90 percentile) using GraphPad Prism. Comparison between groups were performed using Mann–Whitney non-parametric unpaired *t* tests. *p* values < 0.05 were reported as significant. (**B**) Flow cytometry analysis of the percentage of CD14+ monocytes expressing the CD16 receptor. Peripheral blood monocytes expression of CD16 could be analyzed in 24 RCE patients analyzed in reference to a group of 24 RCE-free patients (RCE−) without a MACE or AHF event, matched for age, gender, and CKD status. Monocyte CD16 expression in RCE patients was further analyzed in a subgroup of 12 MACE+AHF− patients and 12 AHF+ patients (among which 3 were AHF+MACE+ and also experienced a MACE event, and 9 were AHF+MACE− and only experienced AHR during the first-year post ACS). Groups of RCE+, MACE+, and AHR+ patients were analyzed using Mann–Whitney non-parametric unpaired *t* tests and compared to proportion of CD16+CD14+ monocytes observed in gender-, age-, and CKD status-matched groups of AHR−MACE− patients. *p* values < 0.05 were reported as significant. *p* values ≥ 0.05 and <0.2 were indicated as a trend for significance.

We also identified a trend associating hsCRP levels with the proportion of CD14+ circulating monocytes expressing the cognate CD32 receptor encoded by FCGR2A in the group of patients with AHF (*r* = 0.550, *p* = 0.138), while we found no such correlation in the RCE-free patients. Moreover, the mean percentages of CD32+CD14+ circulating monocytes were significantly higher in patients who developed AHF (72%) than in age- and gender-matched patients who did not (40%), see Figure 2A. The proportion of CD16+ monocytes was comparable in RCE+ and RCE-groups, see Figure 2B.

### 3.3. The FCGR2A rs1801274 HH Genotype Associates with the Subsequent Risk of Developing MACEs

**Figure 3:**
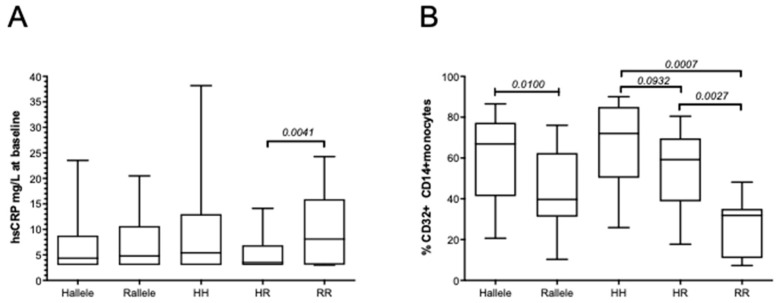
*FCGR2A* polymorphisms are associated with baseline hsCRP levels and CD32 expression in CD14+ monocytes. (**A**) Baseline hsCRP levels were analyzed in group of patients with ACS according to the presence of the H (*n* = 113) or R allelic variants (*n* = 102). HH (*n* = 43), HR (*n* = 70), and RR (*n* = 32) refer to the homozygous of heterozygous combinations of FCGR2A allelic variants at the extracellular amino acid position 131: arginine (R) or histidine (H). Groups of RCE+, MACE+, and AHR+ patients were analyzed using Mann–Whitney non-parametric unpaired *t* tests and compared to the control group of AHR−MACE− patients. *p* values < 0.05 were reported as significant. hsCRP variables are described as box and whiskers plots representing medians and (10–90%) using GraphPad Prism. Comparison between groups were performed using Mann–Whitney non-parametric unpaired *t* tests. *p* values < 0.05 were reported as significant. (**B**) The expression of CD32 on circulating CD14+ monocytes (%CD32+CD14+ cells) were analyzed in a subgroup of 58 patients with ACS according to the presence of the H or R allelic variants. HH, HR, and RR refer to the homozygous of heterozygous combinations of FCGR2A allelic variants at the extracellular amino acid position 131: arginine (R) or histidine (H). Proportion of CD32+ monocytes are described using GraphPad Prism as box and whiskers plots representing medians and (10–90%). Comparison between groups were performed using Mann–Whitney non-parametric unpaired *t* tests. *p* values < 0.05 were reported as significant, and *p* values ≥ 0.05 and <0.2 were indicated as a trend for significance.

**Figure 4:**
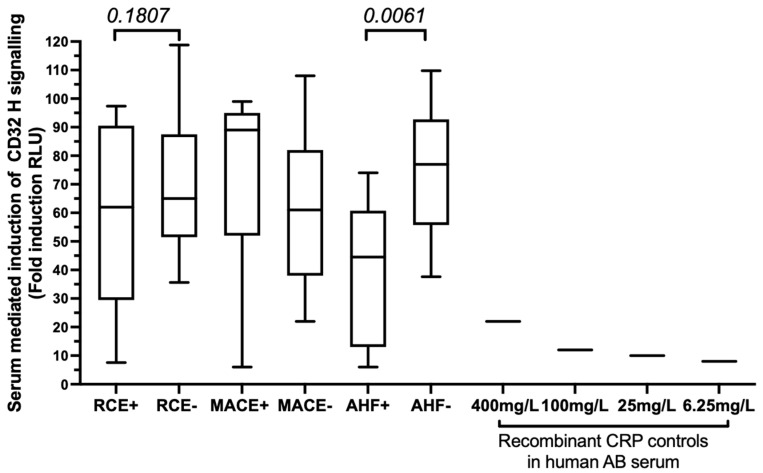
Evaluation of serum-induced activation of CD32-H-dependent activation in patients who bear the *FCGR2A* H allelic variants (HH and H/R genotypes). We assessed the serum-induced ADCP activation potential of serum from patients with the H allele at position 131 of the CD32 protein using a reporter luciferase assay in Jurkat cells transfected with the CD32-H variant. Results are expressed as fold RLU induction analyzed in reference to human AB serum of a non-immunized healthy male control donor. Recombinant CRP was diluted in the human male SAB control serum using different concentration (400 mg/mL, 100 mg/L, 25 mg/L, 6.25 mg/L). Variables that quantify serum-induced ADCP were compared in the group of RCE+ (*n* = 15) and REC− patients (*n* = 19) that bear the FCGR2A H allelic variant. The RCE-positive group was further stratified as MACE+AHF− patients (*n* = 7) and AHF+MACE− patients (*n* = 8). Serum-induced ADCP activation is described as box and whiskers plots representing medians and (10–90%) using GraphPad Prism. Comparison between groups were performed using Mann–Whitney non-parametric unpaired *t* tests. *p* values < 0.05 were reported as significant. *p* values ≥ 0.05 and <0.2 were indicated as a trend for significance.

The median baseline hsCRP levels in the patient’s cohort were shown to be lower in patients carrying the *FCGR2A*-HR heterozygous genotype when compared to those carrying the *FCGR2A*-RR genotype (Figure 3A). Accordingly, using logistic regression, we found that high CRP levels (above the 2.6 or 3 mg/L thresholds) were not associated with the *rs1801274* SNP *FCGR2A*-HH and RR homozygous combination but were inversely correlated with the presence of the *FCGR2A*-HR heterozygous gene variants (OR: 0.39, *p* = 0.009, 95% CI, 0.197–0.792). The *FCGR2A*-HH genotype was further associated with the increased CD32 expression in monocytes, and the percentage of CD32+CD14+ monocytes was significantly lower in patients with the *FCGR2A/CD32* R allelic variant (40%) than in patients with the H allele (67%), see Figure 3B. We further used a luciferase reporter assay evaluating the potential of sera from patients bearing the H allele to activate the CD32-dependent ADCP activity of Jurkat cells transfected with the human *FcγRIIa* (H131 variant). Our results revealed that the serum-mediated activation levels were similar in MACE patients who displayed the HH or HR combination but were significantly lower in AHF+ patients with the H allele than in event-free patients with the H allele (Figure 4). This lower CD32H activation potential observed in patients with AHF bearing the H allele was not correlated with the hsCRP levels evaluated in serum or with the CD32 monocyte cell surface expression.

**Figure 5:**
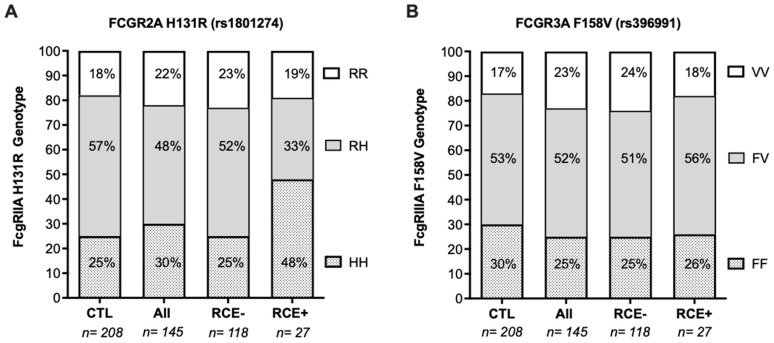
Distribution of FCGR2A rs1801274 and FCGR3A Rs 396,991 genetic allelic variants associated with development of RCE during the first year after initial ACS diagnosis. (**A**) Distribution of the FCGR2A genotype in the whole cohort of patients with ACS (all patients) subdivided into patients who developed RCE and those who remained RCE free (no RCE). HH, HR, and RR refer to the homozygous or heterozygous combinations of FCGR2A allelic variants at the extracellular amino acid position 131: arginine (R) or histidine (H). Frequencies in patient groups were analyzed in reference to those in healthy control groups. (**B**) FF, VF, and VV refer to the combinations of FCGR3A allelic variants at position 158: valine (V) or phenylalanine (F). FF indicates the homozygous phenylalanine genotype; VV indicates the homozygous alanine genotype; VF indicates the heterozygous valine/phenylalanine genotype. RCE, recurrence of cardiovascular events; CTL, healthy control individuals.

The distribution of homozygous and heterozygous gene variants at the *FCGR2A* and *FCGR3A* gene loci were similar in patients with ACS when analyzed in reference to the group of healthy control individuals (Figure 5). We found no association between the *FCGR3A* rs396991 SNP variants encoding CD16 and the occurrence of RCEs in the study cohort, but the proportion of patients with the *FCGR2A* rs1801274 HH homozygous genotype was significantly higher within the group of patients who developed RCE (48.15%) than it was within the group of ACS control individuals who remained event free during the 1-year follow-up (25.42%) (Figure 5; *p* = 0.0197). The distribution of the *FCGR2A* and *FCGR3A* genotype combination observed in the cohort of control healthy individuals was similar to that observed in RCE-free patients after 1 year. The *FCGR2A*-HH genotype was further associated with an increased risk of developing RCE (OR, 2.7; *p* = 0.23; 95% CI, 1.2–6.44), rather suggesting a protective role for the *FCGR2A* R allele. Within the group of patients who developed RCE, univariate logistic regression analyses showed that an associated *FCGR2A*-HH genotype was predictive of MACE recurrences (odds ratio, 3.56; *p* = 0.014; 95% CI, 1.29–9.78) but not of AHF. Our multivariate analysis including other variables identified as RCE risk factors (hypercholesterolemia, LVEF <40) confirmed that the presence of the *FCGR2A*-HH homozygous genotype identified patients at high risk of developing RCE during the first-year post PCI, independently of their high baseline level of hsCRP (Table 3).

## 4. Discussion

Our study is the first one reporting evaluation of the *FCGR2A* polymorphic variants that encode the CD32-activating receptor as potential biomarkers that may help predict early recurrence of adverse cardiovascular events after initial PCI for ACS. Our results suggest that in patients with primary ACS, the *FCGR2A*-HH homozygous genotype can predict the risk of recurrence of RCE during the first post PCI year, independently of the presence of levels of hsCRP above the 3 mg/L threshold, which are commonly associated with poor cardiovascular prognoses. The new evidence of our study is that, in patients with an initial ACS, increased monocyte CD32 expression in circulating cells is associated with the presence of the H allelic variant encoding CD32 and with the subsequent development of AHF.

In contrast to previous studies that indicated that preoperative hsCRP circulating levels can be useful biomarkers for predicting the subsequent risk of MACEs in patients with myocardial infarction and 6-month post-operative cardiovascular events [22], observations in our study cohort identify CRP elevation in patients with primary ACS as a marker of heart failure but not of MACEs following PCI, suggesting that distinct mechanisms may be at play in these two outcomes. Inflammation is considered a central pathophysiological mechanism contributing to the pathogenesis and progression of cardiovascular diseases, and it has been identified both as a cause and consequence of heart failure [23]. As underlined in other reports [23,24,25], it remains hard to distinguish whether enhanced baseline CRP levels are merely the reflection of systemic inflammation associated to risk factors of primary ACS disease diagnosis, such as gender, ageing, obesity, and diabetes, or if it plays an active role in disease progression. Increasing evidence shows that the CRP is not only an inflammatory biomarker associated with common environmental and lifestyle risk factors, but that it is also an important risk factor associated with cardiovascular and other age-related diseases [26,27,28,29]. In the patients with ACS evaluated in this study, according to our univariate regression analysis of circulating hsCRP levels above the 3 mg/L hsCRP threshold, commonly defined as a cardiovascular risk factor associated with poor prognosis in other studies (22), were further associated with the female gender (*p* = 0.007) and with common cardiovascular risk factors such as obesity (*p* = 0.054), diabetes (*p* = 0.047), older age (>65 years; *p* = 0.010), chronic kidney disease (eGFR <45; *p* = 0.034), and increased baseline BNP levels (*p* = 0.001). The upregulation of CRP can also reflect induction of CRP in response to trauma and tissue damage or infectious challenges. Mendelian randomization studies analyzing the genetic association of *CRP* SNPs with cardiovascular diseases demonstrated a significant association between the *CRP* genetic variants and baseline circulating CRP levels and identified CRP levels as a biomarker of inflammation rather than a mediator of CVD [30]. Our multivariate models did not allow for the identification of MACE recurrences based on high CRP levels, instead they described CRP and CD32 monocyte profiles associated with development of AHF. In our study cohort, we observed that increased CRP levels and a high proportion of monocyte-expressing CD32 receptors were common in patients with a history of proinflammatory conditions associated with a higher risk of AHF events during the first year following their initial ACS presentation, such as lower kidney function, anemia, and diabetes. Increased CRP values (>3 mg/L) have also been identified as predictors of heart failure and its severity in high-risk populations [31]. Heart failure is a complex syndrome caused by cardiac abnormalities, which result in the impairment of the ventricular ejection function. As a high hsCRP level has been associated with an increase in proinflammatory cytokines such as IL6, it may sustain chronic inflammation during atherosclerosis progression and heart failure. Accordingly, the high circulating CRP levels in patients with ACS who develop AHF may reflect this IL-6-mediated upregulation of CRP expression. CRP has also been shown to promote cell death and phagocytosis of ischemic/hypoxic cells when underlying acute myocardial infarction [32]. Our finding that CD32-positive circulating monocytes are more abundant in patients who develop AHR are in line with those in other reports that suggested that upregulated CRP levels may promote the differentiation of human CD32 monocytes towards an M1 proinflammatory phenotype [33,34], thereby amplifying myocardial damage and contributing to development of heart failure after an initial non-myocardial infarction ACS. While such proatherogenic CRP effects have been shown to be processed though CD32 receptors on monocytes and aortic endothelial cells, the evidence on the differences in FcγR expression data in monocytes of patients with recurrent cardiovascular events compared with the data of control subjects is sparse [35]. The enhanced expression of CD32 in monocytes that bear the FCGR2A-HH genotype with lower affinity for CRP in our study may indicate that these interactions do not directly target monocyte-driven inflammation and that increased circulating CRP levels reflect the abundant availability of this inflammatory molecule to target other cell types though specific adverse mechanisms that promote AHF rather than MACE. The restriction of our CD32 expression phenotypic analysis to circulating monocytes is a limitation of this study because we could not assess the role of FCGR2A polymorphisms on CRP sensing by other cell types. Indeed, CRP also targets neutrophils, platelets, or other tissue-resident CD32+ cell types that may be relevant to cardiovascular diseases (such as endothelial cells, cardiomyocytes, or fibroblasts that were not explored in our study). CRP has also been shown to contribute to inflammatory fibrotic processes induced by activation of CD32-positive fibroblast-like synoviocytes in patients with rheumatoid [36]. CRP can be produced at the vessel wall by vascular smooth muscle cells from human coronary arteries and vulnerable plaques after PCI injury [37], and CRP may activate the oligodeoxynucleotide-binding protein (Mac-1) and promote restenosis [38,39,40].

Our study is the first one presenting an integrated analysis of FCGR2A genetic variants and cell surface expression of the CD32 receptor for CRP in monocytes of patients with recurrent cardiovascular events and in matched control subjects. We provide novel evidence showing that high proportions of circulating CD32+ monocytes are associated with expression of the CD32 H allele and recurrence of AHF (but not of MACE), suggesting that the FCGR2A genetic background may also sustain the persistence of CRP-mediated inflammatory signals in patients after an initial ACS episode. Interestingly, the FCGR2-HH susceptibility genotype associated with MACEs has been described to encode a receptor with lower affinity for CRP [36], a finding that is difficult to reconciliate with the fact that it may be associated with a higher pro-inflammatory status resulting from CRP-triggered CD32-dependent monocyte activation. Indeed, our results after testing of the CD32HH-dependent activation signalling using the reporter assay suggest that serum from HH patients with ACS who will develop AHF produces low activation of CD32-transfected cells expressing the H allele. Our observations suggest that the H allele may be associated to CRP-independent regulatory mechanisms that favor enhanced expression of the CD32-activating receptors in monocytes of ACS patients, thus suggesting that, after an initial ACS episode, the genetic background of FCGR2A may indirectly regulate CRP levels and associated dammage in a host personalized manner. GWAS studies identified the FCGR2A-131H allelic variant with lower affinity for CRP as a susceptibility marker for Kawasaki disease in male patients [41], a pediatric vasculitis associated with coronary artery aneurysms [42,43]. Homozygosity for FCGR2A-131HH has also been described as a susceptibility marker associated with malarial anemia [44]. These studies suggest that other pathogenic mechanisms that lead to the CD32-mediated triggering of cytokine production may be at play to sustain inflammation after initial ACS. The rs1801274 SNP in *FCGR2A* has been reported to affect the affinity of FCGR2A for IgG subclasses and CRP, thus regulating immune complexes’ mediated phagocytosis and the release of inflammatory mediators [45] in response to infectious or inflammatory conditions associated to autoimmune and cardiovascular risk factors. As the His131 allelic variant with lower affinity for CRP binds IgG2 and IgG3 with greater affinity than the Arg131 allelic variant [8,14,46,47], one cannot exclude that CRP and IgG ligands exert competitive/antagonist signaling on CD32-mediated activation of monocytes as a result of underlying chronic humoral activation. The association of the FCGR2A-131HH genotype to MACE and RCE could thus reflect indirect effects associated to the presence of auto-antibodies that result in triggering of CD16- and CD32-dependent pro-inflammatory cytokine secretion by monocytes and NK cells, in part independent of CRP levels. A recent report indeed shows that donors with the homozygous *FCGR2A*-HH genotype show enhanced IgG1-triggered FcγRIIa monocyte activation that leads to the release of pro-inflammatory cytokines that in turn induce IFN-γ secretion by NK cells [9]. The same study further revealed that the magnitude of the IFN-γ induction is impacted by the linkage disequilibrium between the *FCGR2A*-131 and *FCGR3A*-158 gene variants. The previously described, LD between rs1801274 *FCGR2A*-131H and rs396991 *FCGR3A*-158-V alleles [39] was also observed in the study cohort and associated the *FCGR3A*-158 V allele to CRP levels greater than 3 mg/L. Thus, the interplay allowing for CRP and IgG interactions with CD16- and CD32-mediated monocyte activation may be indirectly influenced by competitive mechanisms associated with a linkage disequilibrium affecting the CRP and IgG immune inflammatory responses in patients with ACS who bear the *FCGR3A*-VV and *FCGR2A*-HH genotypes; this possibility needs to be investigated in large cohorts.

The limitations of our study are related to the size and heterogeneity of the ACS patients enrolled and to the fact that analysis of CD32 expression was restricted to monocytes; however, our results warrant further large-scale validation studies evaluating CRP and CD32 profiles to ensure that these inflammatory ligand receptor pathways can be of value to anticipate the individual inflammatory risk of persistent inflammation after an initial PCI.

## 5. Conclusions

Our findings serve as preliminary evidence that host *FCGR2A* genetic variants can influence individual monocyte CD32 receptor expression variability, and may thereby contribute to the fine-tuning of CD32-driven chronic activating signals that affect the risk of developing RCEs following primary ACS events. Considering the well-documented association between circulating levels of hsCRP and cardiovascular disorders that prompted the routine evaluation of this inflammatory biomarker in clinical settings [44], further investigations of susceptibility markers that characterize the specific features of the CD32 receptor may serve to refine the early stratification of ASC patients with high CRP levels at risk for AHF versus those at risk for MACE to optimize their management with individualized life style recommendations and targeted approaches aimed at reducing inflammation.

## Figures and Tables

**Table 1:** Angiographic and interventional characteristics of the study population.

Variables	All Patients *n* = 145	RCE+ *n* = 27	RCE− *n* = 118	*p* Value RCE vs. RCE−
**Procedural characteristics**				
Number of treated lesions	1 (1–2)	1 (1–2)	1 (1–1.75)	ns
Radial access, % (n)	96% (138)	93% (25)	97% (113)	ns
Number of diseased vessels	3 (2–4)	3 (2–4)	3 (2–4)	ns
Monotroncular, % (n)	34% (49)	34% (9)	33% (40)	ns
Bitroncular, % (n)	30% (44)	31% (8)	30% (36)	ns
Tritroncular, % (n)	36% (52)	36% (10)	37% (42)	ns
Mean Stent diameter (mm)	3 (2.75–3.5)	2.95 (2.71–3)	3 (2.75–3.5)	ns
Number of stents	1 (1–2)	1 (1–2)	1 (1–2)	0.0947 ^t^
Total stent length (mm)	24 (17.5–32.2)	26.5 (20.5–41.5)	23 (16–32)	0.0558 ^t^
**Diseased vessels**				
Left anterior descending coronary artery	85.5 % (124)	85% (23)	86% (101)	ns
Circumflex artery	50% (73)	63% (17)	47% (56)	ns
Right coronary artery	60% (87)	52% (14)	62% (73)	ns
Left main coronary artery	8% (12)	7.4% (2)	8.5% (10)	ns
Left ventricular ejection fraction	60 (45.7–64)	47 (36–55)	60 (50–65)	0.0003

Values indicated refer to median (first–third quartile) or % (number of patients). ns, non-significant. Significant *p* values were noted using asterisk: Trends: *p* value >0.05 and <0.2 are noted with ^t^.

**Table 2:** Patients’ characteristics.

	All Patients *n* = 145	RCE+ *n* = 27	RCE− *n* = 118	*p* Value RCE+ vs. RCE−
Age (years)	61 (55–71)	65 (57–71)	60 (54–71)	ns
Male gender %	77.24	66.67	79.66	ns
Body mass index (BMI), kg/m^2^	27.18 (24–31)	27 (25–31)	27 (24–31)	ns
**Clinical presentation**				
Non STEMI %	73.79	88.89	70.34	ns
STEMI %	20	11.11	22.03	ns
Unstable angina %	6.21	0	7.63	ns
**Cardiovascular risk factors**				
Active smoker	36.55	25.93	38.98	ns
Hypercholesterolemia	37.24	59.26	32.2	0.0087 **
Hypertension	58.62	55.56	59.32	ns
Obesity	28.3	25.9	28.8	ns
Heredity	8.97	14.81	7.63	ns
Diabetes Mellitus	43.45	62.96	38.98	0.0234 *
**Medical history**				
Myocardial infraction (MI)	27.08	37.04	24.79	ns
Percutaneous coronary intervention	37.93	48.15	35.59	ns
Coronary artery bypass graft	5.52	7.41	5.08	ns
Stroke %	5.5	7.4	5	ns
Chronic kidney disease %	5.52	14.8	3.4	0.0398 *
Peripheral arterial disease %	8.97	22.2	6	0.0164 *
**Clinico-biological parameters at time of enrolment (T0)**				
White blood cell count (Tera/L)	7.45 (6.42–10.19)	7.94 (6.6–10.8)	7.39 (6.4–10.1)	ns
Hemoglobin (g/L)	133 (121–141)	124 (112–138)	135 (123–141)	0.0285 *
Hematocrite (%)	39 (36–42)	37 (33–41)	39 (36–42)	0.0889 ^t^
Platelets (Giga/L)	234 (191–285)	270 (210–332)	226 (186–272)	0.0263 *
Creatinine (umol/L)	72 (59–87)	79 (63–113)	70 (59–85)	0.0742 ^t^
eGFR (mL/min/1.73 m^2^)	92 (78–103)	80 (51–96)	95 (81–105)	0.0065 **
CRP (mg/mL)	5 (3–10.7)	5.5 (3.2–15)	4.7 (3–10.6)	0.0066 **
hs CRP	3.97 (1.46–9.84)	4.79 (2.4–12.5)	3.78–1.36–9.53)	0.0067 **
BNP (ng/L)	59.65 (18–138)	190 (94–460)	50 (16–95)	0.0068 **
Troponin	0.12 (0.01–1.8)	0.2 (0.03–2.03)	0.1 (0.01–1.76)	ns
**Medications at discharge**				
Aspirine	95.86%	88.89%	97.46%	0.0784 ^t^
Beta blockers	70.34%	85.19%	66.95%	0.0613 ^t^
Calcium channel blockers	9.66%	11.11%	9.32%	ns
P2Y12 ADP receptor inhibitors	93.8	92.6	94	ns
ACE inhibitors/ARBs	61.4	74.10	58.5	ns
insulin	17.2%	29.63%	14.41%	0.0866 ^t^
Biguanides	6.9%	85.19%	94.92%	0.0904 ^t^
Proton pump inhibitors	82.76%	81.48%	83.05%	ns
Statins	90.34%	96.3%	88.98	ns
**Recurrent cardiovascular events during the first year (RCE)**				
Cardiovascular death	1.4%	7.41%	na	na
Acute myocardial infarction	6.2%	33%	na	na
Urgent revascularization	6.9%	37%	na	na
Stroke	0%	0%	na	na
Acute Heart Failure (AHF)	9%	48%	na	na

RCE+ refer to patients that meet the primary endpoint of Recurrence of Cardiovascular Events (RCE) while RCE− refers to patients that remain free of event during the 1-Year post PCI follow-up. ns refer to *p* values > 0.05, significant pvalues were noted using asterisk: * *p* ≤ 0.05,** *p* ≤ 0.01. Trends: *p* value >0.05 and <0.1 are annoted as ^t^; na, not applicable; results are expressed as median and 25%–75% interquartile ranges or proportion (%). ACE inhibitors, angiotensin-converting-enzyme inhibitors; ARB, angiotensin II receptor blockers.

**Table 3:** Multivariate analysis of variables associated with high RCE risk.

RCE	Univariate Logistic Regression Analysis			Multivariate Logistic Regression Analysis		
Variables	Odds Ratio	*p* Value	95% CI	Odds Ratio	*p* Value	95% CI
**FCgR2A-HH**	2.70	**0.0228**	1.15–6.35	2.71	0.0480	1.01–7.44
**FEVG < 40%**	4.46	**0.0060**	1.56–12.69	4.70	0.0124	1.40–16.2
**Hypercholesterolemia**	3.00	**0.01**	1.3–7.14	4.37	0.0024	1.67–12.42
hs CRP ≥ 3 mg/L	1.91	0.1419	0.81–4.87	2.08	0.1514	0.77–6.14

A bivariate logistic regression allowed us to estimate crude odds ratios with their 95% confidence intervals (95%CI). In addition, we performed a multivariate logistic regression to assess the independent associations of clinico-biological characteristics with RCE. We selected relevant variables from the bivariate analysis (left panel, FCGR2A-HH, FEVG < 40%, and hypercholesterolemia, highlighted in bold based on a threshold *p* value < 0.05), except for the CRP ≥ 3 mg/L that was forced in a multivariate model (right panel) to test the predictive values of other identified parameters independently of CRP. Firth’s correction was applied by performing Firth’s penalized-likelihood logistic regression to consider small numbers. Adjusted odds ratios and their 95% confidence intervals were estimated. Statistical analyses were conducted using R software.

## Data Availability

The raw data supporting the conclusions of this manuscript can be made available by the authors, without undue reservation.
